# Identifying patterns of neuropathological diagnoses in medico-legal autopsies – A latent class analysis of 1270 neuropathologically examined cases from Finland

**DOI:** 10.1007/s12024-026-01195-9

**Published:** 2026-02-27

**Authors:** Xiaojian Keinänen, Paula K. Vauhkonen, Petteri Oura

**Affiliations:** 1 Department of Forensic Medicine, Faculty of Medicine, University of Helsinki, P.O. Box 63, Helsinki, FIN- 00014 Finland; 2 Forensic Medicine Unit, Finnish Institute for Health and Welfare, P.O. Box 30, Helsinki, FIN- 00271 Finland

**Keywords:** Central nervous system, Neuropathological examination, Forensic pathology, Legal medicine, Latent class analysis

## Abstract

Neuropathological diseases and injuries are common causes of death and frequently encountered in medico-legal autopsies. Previous studies have suggested that many neuropathological findings co-occur. This retrospective and register-based study aimed to identify latent subgroups of neuropathological diagnoses, including both disease and injury findings. The sample comprised 1270 neuropathologically examined medico-legal autopsy cases from the Forensic Medicine Unit of the Finnish Institute for Health and Welfare over the years 2016–2022. Neuropathological diagnoses were obtained from neuropathologists’ reports, and background characteristics were collected from medico-legal cause-of-death investigation documents. Latent class analysis (LCA) was applied to identify clustering of neuropathological diagnoses among the sample. The dataset comprised 60.6% males, and the median age at death was 64 (interquartile range 43–77) years. LCA models with 2–8 classes were explored; the 3-class model was considered best fit, based on Bayesian Information Criterion values and interpretability. Class 1 was characterized by high prevalences of neurodegenerative and degenerative cerebrovascular diseases, as well as highest age. Class 2 included most cases with acute haemorrhages, their potential causes, and secondary complications. Class 3 comprised a diverse range of neuropathological diagnoses, including cases with epilepsy-related consultation questions and diagnoses. Awareness of co-occurring neuropathological diagnoses would be expected to advocate diagnostic vigilance and facilitate contextual interpretation of co-existing findings in the medico-legal practice. Having demonstrated the feasibility and potential relevance of LCA in forensic neuropathology, this study encourages future applications of LCA in other medico-legal contexts.

## Key Points

The latent class solution with three classes appeared to fit the dataset best.

Class 1 was characterized by neurodegeneration.

Class 2 was characterized by acute haemorrhagic conditions.

Class 3 comprised a diverse range of neuropathological diagnoses.

## Introduction

Neurological disorders are some of the leading causes of death globally (11.1 million deaths in 2021), and the burden of neuropathological diseases increases as the population ages [1]. Central nervous system conditions leading to death are diverse, including diseases, traumatic injuries, and their secondary complications. As for non-traumatic entities, neurodegenerative diseases, epilepsy, tumours, infections, and cerebrovascular diseases form some of the major neuropathological themes in autopsies [2–6]. As for traumatic entities, contusions and traumatic haemorrhages are among the common findings [7]. It is therefore evident that neuropathological examination may constitute a vital part of medico-legal cause-of-death investigation [6, 8].

Knowledge of the potential co-occurrence of neuropathological entities may help forensic pathologists, neuropathologists and clinicians look for and detect findings that often co-occur, as well as improve the understanding of underlying mechanisms. This would be expected to improve interpretation, support differential diagnosis, and reduce cognitive bias in diagnostic decision-making. As examples of coupled entities, previous studies have shown that neurodegenerative findings, such as Alzheimer’s disease neuropathologic change (ADNC), Lewy body disease, and cerebral amyloid angiopathy often occur together, and the prevalence of comorbidities increases with age [9, 10]. As another example, neuropathological findings associated with epilepsy and seizure activity are diverse, including hippocampal sclerosis, cortical and vascular malformations, cerebellar atrophy, contusions, and oedema [3].

Non-traumatic and traumatic findings can also be expected to co-occur. For example, Alzheimer’s disease, Lewy body disease, and Parkinson’s disease are associated with a higher risk of falls and head injuries [11–13]. In addition, seizures and epilepsy are common complications of traumatic brain injury (TBI) [14], and repeated TBI may also lead to chronic traumatic encephalopathy [15]. In these scenarios, the neuropathological examination of a decedent’s brain may reveal both disease pathology and signs of acute or remote traumatic injuries.

Notably, traumatic injuries often co-occur and accumulate depending on the mechanism of injury. For example, simultaneously occurring findings in fatal road traffic accidents are skull fractures, subdural and subarachnoid haemorrhages [16]. In addition, our research group has recently identified combinations of primary head injuries among Finnish medico-legal autopsy cases using latent class analysis (LCA) [17]; circumstances with assumably higher traumatic forces were associated with the co-occurrence of subarachnoid and intracerebral haemorrhages and contusions in particular. It therefore appears reasonable to assume that distinct subgroups of neuropathological diagnoses may well exist in medico-legal autopsy material.

To the best of our knowledge, the potential of LCA has not yet been fully utilized in the medico-legal context. We believe LCA may serve as a useful link between statistical clustering and forensic reasoning, as it can reveal and highlight characteristic patterns relevant for medico-legal decision-making, even in relatively complex datasets. In this study, we aimed to identify latent subgroups of neuropathological diagnoses, including both disease and injury findings, in a large nationwide sample of Finnish medico-legal autopsy cases over the period of 2016–2022. The hypothesis was that distinct combinations of neuropathological diagnoses would be observed in our sample. While the clustering was based on exploratory premises, it was expected to potentially reflect age-related disease burden and trauma-related patterns in the dataset. We also aimed to explore whether the identified subgroups differed in background characteristics.

## Materials and methods

### Study protocol

In Finland, a medico-legal investigation into the cause and manner of death is legally required in cases where there is lack of medical history that could account for the death; when the death occurs suddenly and unexpectedly; and when there is reason to suspect unnatural causes (accident, suicide, homicide, occupational disease, medical or surgical adverse event, war) [18]. The police is the authority responsible for deciding whether to initiate a medico-legal investigation and order a medico-legal autopsy.

Approximately 15% of all deaths undergo a medico-legal autopsy in Finland [18, 19]. These autopsies are carried out by forensic pathologists working at the regional branches of the Forensic Medicine Unit, which operates under the Finnish Institute for Health and Welfare. There are five regional offices, each serving its designated geographic area (Southern Finland, Southwestern Finland, Western and Inland Finland, Eastern Finland, and Northern Finland including Lapland) [19]. Medico-legal cause-of-death investigation documents, including referrals and statements of ancillary examinations such as neuropathology consultations, are handled and stored in a centralized electronic database.

This retrospective, register-based study focused on medico-legal autopsy cases from the years 2016–2022; inclusion in the study required that a comprehensive neuropathological examination had been performed by a neuropathologist. Data for the study were retrieved from the national electronic information system and included background characteristics and neuropathological diagnoses of the cases.

Approval for the study was granted by the Finnish Institute for Health and Welfare (THL/1802/6.02.00/2023). The study was performed in accordance with the Declaration of Helsinki and national legislation on medical research. Ethical approvals were not required since the study was retrospective and only concerned register-based data.

### Background characteristics

Age (in years) and sex (male or female) were obtained from the autopsy order filed by the police. Information on body height (in centimetres), body weight (in kilograms), and fresh brain weight (in grams) derived from the medico-legal autopsy report, with all measurements taken during the autopsy procedure. The recorded brain weight encompassed the cerebrum, cerebellum, brainstem, and leptomeninges. Postmortem interval was calculated as the difference (in days) from the estimated date of death to the date of autopsy.

Neuropathology consultation themes were extracted from the referral, which was formulated by the forensic pathologist. The consultation themes were classified as follows, reflecting initial forensic questions: TBI, hypoxia-ischaemia, neurodegeneration, epilepsy, and other/unspecified. One referral could involve multiple themes. Manner of death (disease/accident/homicide/suicide/other) was indicated on the death certificate, issued by the forensic pathologist upon completion of the cause-of-death investigation.

### Neuropathological diagnoses

Neuropathological examinations were conducted in local neuropathology departments on brains that had been preserved in formaldehyde. The examinations involved both macroscopic dissection and histological analysis of tissue samples; the extent and specific neuropathological methods were at the discretion of the neuropathologist.

For this study, neuropathological diagnoses were collected from the diagnosis line of the neuropathologist’s consultation report. The following diagnosis variables were formulated:

- Hypoxic-ischaemic neuronal injury (eosinophilia and pyknosis of damaged neurons in haematoxylin-eosin stain)
- Oedema (macroscopic or microscopic)
- Herniation (of any type)
- Aneurysm (of any type)
- Atherosclerosis (in the circle of Willis)
- Arteriolosclerosis (in parenchymal vessels)
- Cerebral amyloid angiopathy (in parenchymal or leptomeningeal vessels)
- Acute infarct (including macroscopic, lacunar, microinfarcts)
- Old infarct (including macroscopic, lacunar, microinfarcts)
- Acute subarachnoid haemorrhage (traumatic or non-traumatic; macroscopic)
- Acute intracerebral haemorrhage (traumatic or non-traumatic; macroscopic or microscopic)
- Acute intraventricular haemorrhage (traumatic or non-traumatic; macroscopic)
- Other/unspecified acute haemorrhage (ill-defined bleeds; traumatic or non-traumatic; macroscopic or microscopic)
- Old haemorrhage (of any type; macroscopic or microscopic)
- Acute contusion/laceration (macroscopic)
- Acute traumatic axonal injury (based on beta-amyloid precursor protein stain)
- Old TBI (of any type)
- Cerebral atrophy (cortical or central)
- Hippocampal atrophy (including hippocampal sclerosis)
- Cerebellar atrophy (including vermal atrophy)
- ADNC (of any severity)
- Lewy body disease (of any stage)
- Frontotemporal lobar degeneration (of any type)
- Motor neuron disease (of any type)
- Other tauopathy (including argyrophilic grain disease, corticobasal degeneration, primary age-related tauopathy, progressive supranuclear palsy)
- Other synucleinopathy (including multiple system atrophy)
- Other Transactive response DNA binding protein 43 kDa (TDP43)-proteinopathy (including limbic-predominant age-related TDP43 encephalopathy)
- Hydrocephalus
- Benign neoplasm (including cyst, dysembryoplastic neuroepithelial tumour, ependymoma, ganglioglioma, germinoma, lipoma, meningioma, neurocytoma, pilocytic astrocytoma, pineal parenchymal tumour, pituitary adenoma, schwannoma, teratoma, xanthogranuloma)
- Malignant neoplasm (including carcinoma, high-grade glioma, lymphoma)
- Infection (including encephalitis, fungus, herpes, meningitis, septic encephalopathy, syphilis, tuberculosis)
- Inflammation (including granulomatous inflammation, encephalitis)
- Malformation of cortical development (including focal cortical dysplasia, cortical microdysgenesis, heterotopia)
- Other malformation (including aqueductal stenosis, Chiari, hippocampal anomaly, holoprosencephaly, vascular malformation)
- Demyelinating disease (including multiple sclerosis)

Diagnoses were taken as they appeared in the diagnosis line of the neuropathologist’s report; there were no limitations based on the severity or extent of a finding, as long as it was formally listed in the diagnosis line. Other neuropathologist’s observations, which were only mentioned in the narrative portion of the report without being included in the diagnosis line, were not included. These constraints should be taken into account when interpreting the class assignments in LCA.

### Statistical analysis

Latent subgroups of neuropathological diagnoses were explored by means of LCA. LCA is a statistical method used to categorize cases into distinct, data-driven subgroups (referred to as classes) based on selected variables of interest [20, 21]. In accordance with the aim of the study, LCA was applied exclusively to neuropathological diagnoses, deliberately excluding all background variables from the analysis; background variables were only analysed post hoc to aid characterize the identified classes.

LCA was conducted in R Studio version 4.4.0 for Windows [22], utilizing the *poLCA* package. Models with 2–8 classes were explored, and fit statistics were recorded for each model. Cases were assigned to the class for which they had the highest posterior membership probability as determined by the model.

The following fit statistics were recorded from the *poLCA* output: Bayesian information criterion (BIC), Akaike information criterion (AIC), likelihood ratio/deviance statistic (G^2^), log-likelihood, and Chi-square goodness of fit (X^2^). BIC served as the primary indicator of model fit (lower values indicating better fit); it was considered superior to AIC in the context of the present study [23]. Of note is the fact that log-likelihood values are expected to improve with increasing model complexity, whereas BIC penalizes the addition of parameters, favoring models that achieve an optimal balance between goodness of fit and parsimony. In addition to fit statistics, the relative sizes of each class and average posterior probabilities of class memberships were also recorded. The model deemed most appropriate for the present dataset was selected as the final solution [21].

Descriptive statistics of categorical variables were presented as percentages and frequencies; the distributions of continuous variables were described using medians and interquartile ranges (IQRs). Statistical comparisons between classes were conducted using the Fisher-Freeman-Halton Exact Test (categorical variables) and the Mann-Whitney U Test (continuous variables). P values < 0.05 were considered statistically significant. Aside from the LCA, all statistical analyses were carried out in SPSS Statistics version 29 (IBM, Armonk, NY, USA).

Bar charts illustrating the prevalence of neuropathological diagnoses within the classes were generated using Excel for Windows version 2506 (Microsoft, Redmond, WA, USA).

## Results

The sample comprised 1270 neuropathologically examined medico-legal autopsy cases from the years 2016–2022. Table 1 shows the background characteristics of the sample. Most cases involved male decedents (60.6%), and the median age at death was 64 (IQR 43–77) years. Median postmortem interval was 7 days (IQR 5–10). The most common neuropathology consultation themes were TBI (39.5%), epilepsy (15.9%) and neurodegeneration (13.7%). The most prevalent manners of death were disease (60.6%) and accident (23.0%). Table 2 shows the neuropathological diagnoses of the sample. The most common diagnoses were hypoxic-ischaemic neuronal injury (28.6%), arteriolosclerosis (23.0%) and ADNC (16.1%).

**Table 1:** Background characteristics of the sample

Characteristic	Full sample (*n* = 1270)	By class			
		Class 1 (*n* = 320)	Class 2 (*n* = 190)	Class 3 (*n* = 760)	*P* value
Sex					
Male	60.6 (770)	51.6 (165)	66.3 (126)	63.0 (479)	
Female	39.4 (500)	48.4 (155)	33.7 (64)	37.0 (281)	< 0.001
Age* (y)	64 (43–77)	80 (72–87)	63 (46–75)	56 (30–70)	
< 20	10.0 (127)	0.0 (0)	8.9 (17)	14.5 (110)	
20–39	12.9 (164)	1.3 (4)	10.5 (20)	18.4 (140)	
40–59	19.8 (251)	5.0 (16)	26.8 (51)	24.2 (184)	
60–79	36.6 (465)	42.8 (137)	36.8 (70)	33.9 (258)	
≥ 80	20.7 (263)	50.9 (163)	16.8 (32)	8.9 (68)	< 0.001
Postmortem interval* (d)	7 (5–10)	8 (6–11)	7 (5–10)	7 (4–10)	< 0.001
Autopsy measurements					
Height* (cm)	170 (160–177)	166 (158–175)	173 (164–178)	170 (160–178)	< 0.001
Weight* (kg)	71 (55–86)	66 (54–80)	73 (61–88)	73 (56–88)	< 0.001
Fresh brain weight* (g)	1420 (1300–1538)	1385 (1283–1485)	1460 (1349–1545)	1424 (1290–1550)	< 0.001
Neuropathology consultation theme (multiple options)					
Traumatic brain injury	39.5 (502)	40.0 (128)	65.3 (124)	32.9 (250)	< 0.001
Hypoxia-ischaemia	11.3 (144)	8.4 (27)	7.4 (14)	13.6 (103)	0.009
Neurodegeneration	13.7 (174)	29.4 (94)	1.6 (3)	10.1 (77)	< 0.001
Epilepsy	15.9 (202)	6.3 (20)	10.0 (19)	21.4 (163)	< 0.001
Other/unspecified	34.8 (442)	30.0 (96)	30.0 (57)	38.0 (289)	0.013
Manner of death					
Disease	60.6 (769)	68.8 (220)	42.1 (80)	61.7 (469)	
Accident	23.0 (292)	22.5 (72)	34.7 (71)	19.6 (149)	
Homicide	3.1 (40)	0.9 (3)	7.4 (14)	3.0 (23)	
Suicide	2.6 (33)	2.5 (8)	1.1 (2)	3.0 (23)	
Other	10.7 (136)	5.3 (17)	12.1 (23)	12.6 (96)	< 0.001

Values are percentages and frequencies, except the rows marked with an asterisk (*) show medians and interquartile ranges

**Table 2:** Breakdown of neuropathological diagnoses among the sample

Neuropathological diagnosis	Full sample (*n* = 1270)	By class			
		Class 1 (*n* = 320)	Class 2 (*n* = 190)	Class 3 (*n* = 760)	*P* value
Hypoxic-ischaemic neuronal injury	28.6 (363)	12.5 (40)	27.4 (52)	35.7 (271)	< 0.001
Oedema	14.4 (183)	1.9 (6)	28.9 (55)	16.1 (122)	< 0.001
Herniation	4.2 (53)	0.0 (0)	20.5 (39)	1.8 (14)	< 0.001
Vascular					
Aneurysm	2.8 (35)	0.3 (1)	12.6 (24)	1.3 (10)	< 0.001
Atherosclerosis	14.1 (179)	29.1 (93)	10.0 (19)	8.8 (67)	< 0.001
Arteriolosclerosis	23.0 (292)	53.4 (171)	15.3 (29)	12.1 (92)	< 0.001
Cerebral amyloid angiopathy	7.7 (98)	28.1 (90)	3.2 (6)	0.3 (2)	< 0.001
Acute infarct	9.6 (122)	12.8 (41)	17.4 (33)	6.3 (48)	< 0.001
Old infarct	11.0 (140)	27.8 (89)	4.7 (9)	5.5 (42)	< 0.001
Acute haemorrhage					
Subarachnoid	11.7 (148)	11.3 (36)	56.3 (107)	0.7 (5)	< 0.001
Intracerebral	5.0 (64)	7.5 (24)	21.1 (40)	0.0 (0)	< 0.001
Intraventricular	2.6 (33)	2.8 (9)	12.6 (24)	0.0 (0)	< 0.001
Other/unspecified haemorrhage	7.2 (92)	6.9 (22)	30.0 (57)	1.7 (13)	< 0.001
Old haemorrhage	2.5 (32)	4.7 (15)	2.1 (4)	1.7 (13)	0.025
Acute traumatic brain injury					
Contusion/laceration	5.2 (66)	3.8 (12)	28.4 (54)	0.0 (0)	< 0.001
Traumatic axonal injury	6.2 (79)	5.0 (16)	20.5 (39)	3.2 (24)	< 0.001
Old traumatic brain injury	4.8 (61)	5.0 (16)	2.6 (5)	5.3 (40)	0.323
Atrophy					
Cerebral	3.9 (50)	1.3 (4)	0.0 (0)	6.1 (46)	< 0.001
Hippocampal	5.4 (68)	6.9 (22)	1.1 (2)	5.8 (44)	0.004
Cerebellar	6.5 (82)	1.6 (5)	7.4 (14)	8.3 (63)	< 0.001
Neurodegeneration					
Alzheimer’s disease neuropathologic change	16.1 (204)	58.4 (187)	1.1 (2)	2.0 (15)	< 0.001
Lewy body disease	5.2 (66)	12.5 (40)	0.0 (0)	3.4 (26)	< 0.001
Frontotemporal lobar degeneration	1.4 (18)	0.0 (0)	0.0 (0)	2.4 (18)	< 0.001
Motor neuron disease	1.7 (22)	1.9 (6)	0.0 (0)	2.1 (16)	0.105
Other tauopathy	2.6 (33)	1.9 (6)	0.0 (0)	3.6 (27)	0.006
Other synucleinopathy	0.6 (8)	0.6 (2)	0.0 (0)	0.8 (6)	0.693
Other TDP43-proteinopathy	1.1 (14)	4.4 (14)	0.0 (0)	0.0 (0)	< 0.001
Hydrocephalus	1.3 (16)	0.3 (1)	2.6 (5)	1.3 (10)	0.060
Neoplasm					
Benign	5.4 (69)	4.4 (14)	3.2 (6)	6.4 (49)	0.135
Malignant	2.2 (28)	0.6 (2)	6.8 (13)	1.7 (13)	< 0.001
Infection	1.3 (16)	1.3 (4)	1.6 (3)	1.2 (9)	0.826
Inflammation	0.9 (12)	0.0 (0)	1.6 (3)	1.2 (9)	0.064
Malformation of cortical development	1.0 (13)	0.0 (0)	0.0 (0)	1.7 (13)	0.008
Other malformation	2.3 (29)	0.0 (0)	3.7 (7)	2.9 (22)	< 0.001
Demyelinating disease	1.0 (13)	0.0 (0)	0.0 (0)	1.7 (13)	0.008

Values are percentages and frequencies. TDP43 = Transactive response DNA binding protein 43 kDa

**Fig. 1:**
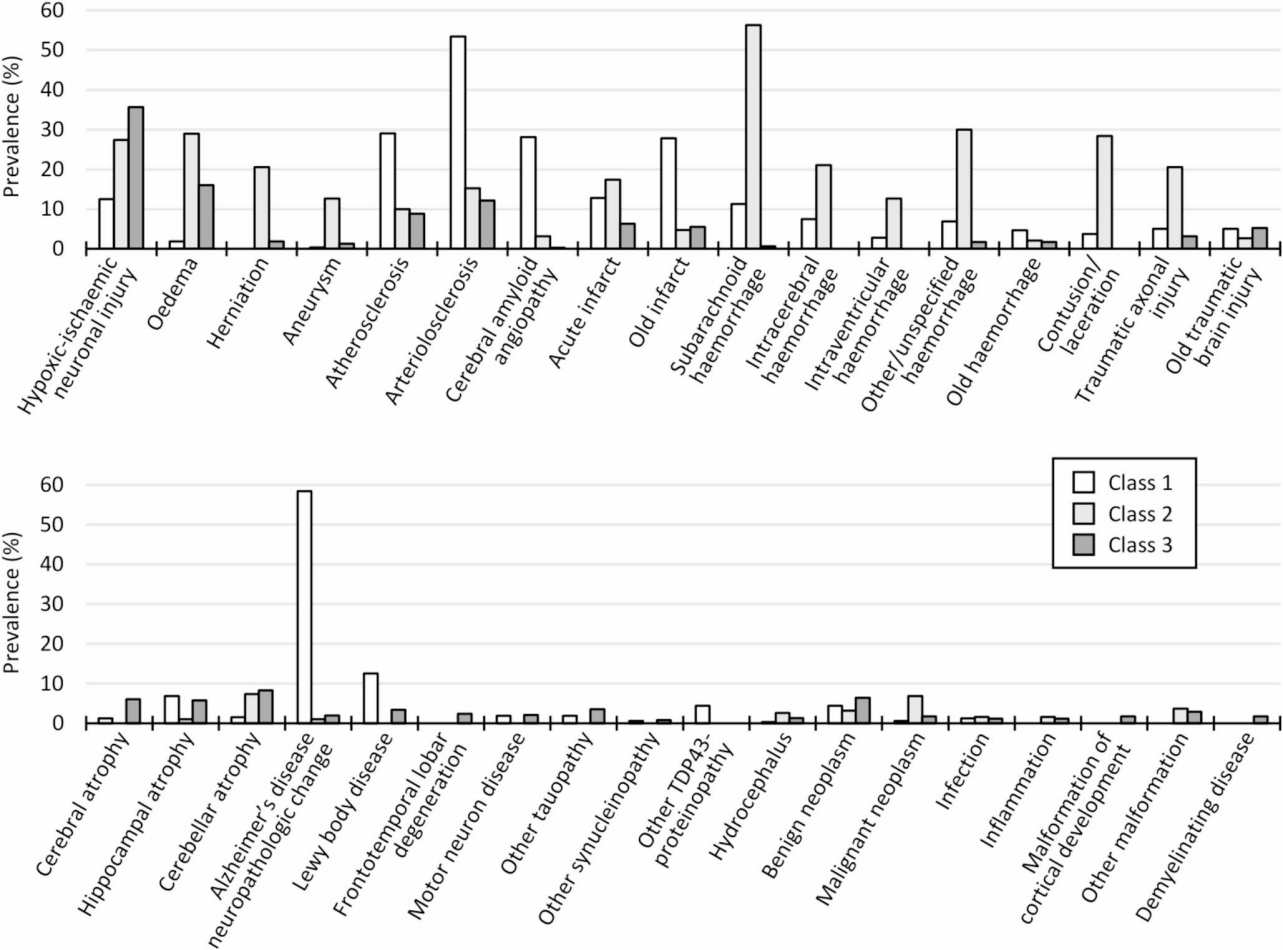
Distribution of neuropathological diagnoses in the latent class model with three classes. TDP43 = Transactive response DNA binding protein 43 kDa

Seven different LCA models were explored (Table 3). The 3-class solution had the lowest BIC value, sufficient class sizes, and high average posterior probabilities. Models with a higher number of classes showed minor gains in log-likelihood but did not justify the increased complexity. Moreover, the 3-class model provided clinically interpretable classes, supporting its selection as the final model. Therefore, the 3-class solution was considered best fit for the present dataset, and was taken forward for further evaluation. As demonstrated in Tables 1 and 2, and Fig. 1, each of the three classes showed distinct features, which are outlined below.

**Table 3:** Fit statistics of the latent class models

Number of classes	BIC	AIC	G^2^	X^2^	Log-likelihood	Class sizes	Average posterior probabilities
2	18542.6	18177.2	4199.4	3,681,835	-9019.6	0.35/0.65	0.86/0.86
3	18459.7	17908.9	3859.1	3,231,693	**-**8847.5	0.25/0.15/0.60	0.83/0.86/0.85
4	18629.1	17893.0	3771.2	2,664,206	-8803.5	0.59/0.15/0.16/0.10	0.86/0.84/0.79/0.73
5	18724.8	17803.3	3609.6	1,052,063	-8722.7	0.16/0.22/0.04/0.15/0.43	0.84/0.77/0.82/0.80/0.79
6	18925.3	17818.5	3552.8	2,996,600	-8694.3	0.08/0.02/0.09/0.15/0.43/0.24	0.86/0.91/0.87/0.84/0.77/0.78
7	19220.3	17928.3	3590.5	1,739,651	-8713.1	< 0.01/0.13/0.42/0.26/0.13/0.03/0.02	1.00/0.84/0.74/0.79/0.77/0.84/0.94
8	19198.3	17720.9	3311.2	402,416	-8573.5	0.13/0.36/0.02/0.19/0.06/0.06/0.13/0.05	0.80/0.72/0.88/0.72/0.89/0.83/0.80/0.95

Class 1 (*n* = 320) was characterized by high prevalences of common neurodegenerative and degenerative cerebrovascular diagnoses. Class 1 had highest median age (80 years) and highest proportion of females (48.4%). The brains were lighter than in other classes (median 1385 g). Most consultations related to neurodegeneration were concentrated in class 1 (29.4%). Disease was the most prevalent manner of death (68.8%). The most frequent neuropathological diagnoses were ADNC (58.4%), arteriolosclerosis (53.4%), atherosclerosis (29.1%), cerebral amyloid angiopathy (28.1%), and old infarct (27.8%). Most Lewy body disease (12.5%), hippocampal atrophy (6.9%), and old haemorrhage (4.7%) diagnoses were also clustered in class 1.

Class 2 (*n* = 190) was characterized by high prevalences of acute haemorrhages and their potential causes (both traumatic and non-traumatic ones), together with secondary changes of space-occupying lesions. Class 2 had highest proportion of males (66.3%), and median age (63 years) was between that of class 1 and class 3. TBI was the most common consultation theme (65.3%), and the prevalences of accidental deaths (34.7%) and homicides (7.4%) were highest of all classes. The most frequent neuropathological diagnoses in class 2 were different types of acute haemorrhages, subarachnoid being the most common (56.3%). Traumatic findings, such as contusions/lacerations (28.4%) and traumatic axonal injuries (20.5%), also prevailed. In addition, there were disease-related findings, such as acute infarcts (17.4%), aneurysms (12.6%), malignant neoplasms (6.8%), and other malformations (3.7%). Finally, secondary findings such as hypoxic-ischaemic neuronal injury (27.4%), oedema (28.9%), and herniation (20.5%) were common in class 2.

Class 3 (*n* = 760) was characterized by diverse neuropathological diagnoses, including lesions potentially associated with epilepsy, and a high prevalence of non-specific findings. Class 3 had the highest number of cases, and also the widest age distribution. Epilepsy was a common consultation theme together with TBI (21.4% and 32.9%, respectively). Disease (61.7%) and accident (19.6%) were the leading manners of death. The non-specific diagnoses hypoxic-ischaemic neuronal injury (35.7%) and oedema (16.1%) constituted the most frequent ones. As for primary pathologies, all cases with frontotemporal lobar degeneration (2.4%), demyelinating disease (1.7%), and malformation of cortical development (1.7%) were clustered in class 3, as were the vast majority of cases with cerebral (6.1%) and cerebellar atrophy (8.3%).

## Discussion

The dataset comprised 1270 neuropathologically examined medico-legal autopsy cases from Finland over the period of 2016–2022. LCA was applied to identify latent subgroups of neuropathological diagnoses, including both diseases and injuries, among the sample. The latent class solution with three classes showed best fit for the present dataset. Each class had a distinct case profile. Class 1 was characterized by neurodegenerative and degenerative cerebrovascular diseases. Class 2 was characterized by acute haemorrhages, their potential causes, and secondary complications. Class 3 comprised a diverse range of neuropathological diagnoses, including cases with epilepsy-related consultation questions and diagnoses.

Most of the neurodegenerative and degenerative cerebrovascular diagnoses, as well as old infarcts and hippocampal atrophy were clustered in class 1. This class had the highest median age (80 years) and proportion of females (48.4%), which may explain the high prevalences of natural deaths and neurodegeneration, especially ADNC in this class [1, 2]. The lowest brain weight and high prevalence of hippocampal atrophy in this class are also likely to be explained by neurodegeneration [2], infarcts and cerebrovascular diseases [24, 25]. Previous studies have indicated that cerebrovascular diseases, especially cerebral amyloid angiopathy, may contribute to Alzheimer’s disease and cognitive decline [26, 27]. Thus, neurodegeneration, degenerative cerebrovascular diseases, and old infarcts were common co-pathologies in our sample, among older individuals in particular.

Class 2 had high prevalences of acute haemorrhages as well as suspected and diagnosed TBI. Accidents and homicides were common manners of death. In general, TBI as well as homicidal and accidental deaths are more common among men, which may explain the high proportion of males in this class (66.3%) [7, 28, 29]. The manifestations of TBI are often haemorrhagic, destructive and space-occupying processes within the cranial cavity, potentially explaining the high rates of secondary complications such as hypoxic-ischaemic neuronal injury, oedema, herniation, and acute infarct. However, there were also a number of non-traumatic causes of haemorrhages, including aneurysms, malign neoplasms and other malformations such as vascular malformations clustered in class 2 [5]. As reported previously, subarachnoid haemorrhages are frequently encountered in medico-legal autopsies, and their aetiologies vary from traumatic to non-traumatic [30]. In our dataset, TBI and acute haemorrhages, regardless of aetiology, were clustered in class 2. LCA did not yield a more nuanced breakdown of the diagnoses within this class, which could relate to constraints in how diagnoses were recorded from the neuropathology report.

In contrast to classes 1 and 2, the neuropathological diagnoses clustered in class 3 were diverse. Class 3 had the highest number of cases (*n* = 760) and widest age distribution. The diagnoses of hypoxic-ischaemic neuronal injury and oedema, both non-specific ones, were common. Interestingly, epilepsy stood out as a common consultation theme, and class 3 manifested several neuropathological diagnoses associated with epilepsy and seizure activity, such as malformation of cortical development, demyelinating disease, tumour, hippocampal and cerebellar atrophy, and old TBI [3, 31]. It therefore seems that decedents with known epilepsy, as well as deaths suspected to be related to epilepsy, were mostly clustered in class 3. Epilepsy-related cases appear to represent a distinct consultation pattern in forensic neuropathology. As always, larger datasets or additional variables might allow for further refinement of subgroups within class 3.

The main strengths of this study were the large sample size and nationwide coverage of all neuropathologically examined medico-legal autopsy cases from a period of 7 years. LCA provided an objective approach to explore the combinations of neuropathological diagnoses from a purely statistical point of view. We hope to have shown the potential of LCA in the context of medico-legal cause-of-death investigation. We are only aware of one previous application of LCA in the context of forensic neuropathology, and it was limited to TBI cases in a much smaller sample [17]. At a higher conceptual level, recognition of co-occurring findings and patterns could well expedite contextual interpretation and benefit the medico-legal field (e.g., cause of death, mechanism of injury, differentiation between traumatic and non-traumatic entities).

A limitation of the present study was the fact that not all medico-legal autopsy cases were associated with a neuropathological examination. As such, there was inevitable selection bias in our data, relative to all medico-legal autopsies and all deaths occurring in Finland. As multiple neuropathologists have served as consultants for the cases, there may have been some variation in diagnostic methods and approaches across the regional neuropathology departments. In the neuropathological diagnosis data, there were no limitations based on the severity or extent of a finding, as long as it was formally listed in the diagnosis line. In haemorrhages, a distinction could not be made between varying aetiologies, as aetiologies were rarely addressed by the neuropathologist in the diagnosis line. Finally, we did not have data on other specific diagnoses of the medico-legal autopsy apart from neuropathology.

## Conclusion

We applied LCA to identify and characterize latent subgroups of neuropathological diagnoses, covering both traumatic and non-traumatic entities, in a large dataset of 1270 Finnish medico-legal autopsies. An LCA solution with three classes was chosen as the best fit for the present dataset. Class 1 was characterized by older age and higher proportion of females, as well as high prevalences of neurodegenerative and degenerative cerebrovascular diseases. Class 2 was characterized by acute haemorrhages, their potential causes, and secondary complications. Class 3 comprised a diverse range of neuropathological diagnoses, including cases with epilepsy-related consultation questions and diagnoses. Knowledge of co-occurring neuropathological findings would be expected to advocate diagnostic vigilance and facilitate contextual interpretation in medico-legal cases. Finally, it appears that the potential of LCA has not yet been fully utilized; having demonstrated the feasibility and potential relevance of LCA in forensic neuropathology, this study encourages future applications of LCA in other medico-legal contexts.
